# Supplementary material for: Dietary acrylamide and physical performance tests: A cross-sectional analysis
Source: PLoS One. 2021 Nov 2;16(11):e0259320. doi: 10.1371/journal.pone.0259320 (PMC8562801; doi:10.1371/journal.pone.0259320)
Supplement: Attachment — Submitted filename: Answers to reviewers.docx [file pone.0259320.s001.docx]

1. Please ensure that your manuscript meets PLOS ONE's style requirements, including those for file naming. The PLOS ONE style templates can be found at

<https://journals.plos.org/plosone/s/file?id=wjVg/PLOSOne_formatting_sample_main_body.pdf> and

<https://journals.plos.org/plosone/s/file?id=ba62/PLOSOne_formatting_sample_title_authors_affiliations.pdf>

**R: The name of the files are in the correct form.**

2. In your Methods section, please provide additional information about the participant recruitment method and the demographic details of your participants. Please ensure you have provided sufficient details to replicate the analyses such as:

a) a description of any inclusion/exclusion criteria that were applied to participant selection,

b) a statement as to whether your sample can be considered representative of a larger population

**R: Added this information.**

3. Thank you for stating the following financial disclosure:

“The funders had no role in study design, data collection and analysis, decision to publish, or preparation of the manuscript.”

At this time, please address the following queries:

a) Please clarify the sources of funding (financial or material support) for your study. List the grants or organizations that supported your study, including funding received from your institution.

b) State what role the funders took in the study. If the funders had no role in your study, please state: “The funders had no role in study design, data collection and analysis, decision to publish, or preparation of the manuscript.”

c) If any authors received a salary from any of your funders, please state which authors and which funders.

d) If you did not receive any funding for this study, please state: “The authors received no specific funding for this work.”

Please include your amended statements within your cover letter; we will change the online submission form on your behalf.

**R: Added, as suggested**.

4. Thank you for stating the following in the Acknowledgments Section of your manuscript:

“The OAI is a public-private partnership comprised of five contracts (N01-AR-2-2258; N01-AR-2-2259; N01-AR-2-2260; N01-AR-2-2261; N01-AR-2-2262) funded by the National Institutes of Health, a branch of the Department of Health and Human Services, and conducted by the OAI Study Investigators. Private funding partners include Merck Research Laboratories; Novartis Pharmaceuticals Corporation, GlaxoSmithKline; and Pfizer, Inc. Private sector funding for the OAI is managed by the Foundation for the National Institutes of Health. This manuscript was prepared using an OAI public use data set and does not necessarily reflect the opinions or views of the OAI investigators, the NIH, or the private funding partners.”

We note that you have provided additional information within the Acknowledgements Section that is not currently declared in your Funding Statement. Please note that funding information should not appear in the Acknowledgments section or other areas of your manuscript. We will only publish funding information present in the Funding Statement section of the online submission form.

Please remove any funding-related text from the manuscript and let us know how you would like to update your Funding Statement. Currently, your Funding Statement reads as follows:

“The funders had no role in study design, data collection and analysis, decision to publish, or preparation of the manuscript.”

Please include your amended statements within your cover letter; we will change the online submission form on your behalf.

**R: Removed the funding information from the manuscript, leaving only in the cover letter. No funding was received by the Authors for this work.**

5. We note that you have stated that you will provide repository information for your data at acceptance. Should your manuscript be accepted for publication, we will hold it until you provide the relevant accession numbers or DOIs necessary to access your data. If you wish to make changes to your Data Availability statement, please describe these changes in your cover letter and we will update your Data Availability statement to reflect the information you provide.

**R: The data of the OAI are freely accessible at:** [**https://www.niams.nih.gov/grants-funding/funded-research/osteoarthritis-initiative**](https://www.niams.nih.gov/grants-funding/funded-research/osteoarthritis-initiative)**, after making a simple login. Therefore, we will not change what declared before.**

6. PLOS requires an ORCID iD for the corresponding author in Editorial Manager on papers submitted after December 6th, 2016. Please ensure that you have an ORCID iD and that it is validated in Editorial Manager. To do this, go to ‘Update my Information’ (in the upper left-hand corner of the main menu), and click on the Fetch/Validate link next to the ORCID field. This will take you to the ORCID site and allow you to create a new iD or authenticate a pre-existing iD in Editorial Manager. Please see the following video for instructions on linking an ORCID iD to your Editorial Manager account: <https://www.youtube.com/watch?v=_xcclfuvtxQ>

**R: Done.**

7. Please review your reference list to ensure that it is complete and correct. If you have cited papers that have been retracted, please include the rationale for doing so in the manuscript text, or remove these references and replace them with relevant current references. Any changes to the reference list should be mentioned in the rebuttal letter that accompanies your revised manuscript. If you need to cite a retracted article, indicate the article’s retracted status in the References list and also include a citation and full reference for the retraction notice.

**R: The reference list is correct.**

Reviewer #1: I reviewed the "Dietary Acrylamide and Physical Performance Tests: A Cross-Sectional Analysis" entitled manuscript and my suggestions are listed below.

General comments: Acrylamide is an important contaminant, and the amount of acrylamide that people take in diet is very important for public health. The topic of the paper is interesting.

**R: We would like to sincerely thank the Reviewer for her/his appreciation for our manuscript. We have tried to further improve it through the comments of Reviewer 1 and 2.**

References should be written according to the journal rules. Punctuation marks should be corrected in the writing of references in the text. At the end of the sentence, the reference should be shown in parentheses, followed by a period. Abbreviations and explanations should be given as footnotes in the tables.

**R: We have addressed all these important points, as suggested.**

In which group is acrylamide classified by the International Agency for Research on Cancer? This information and metabolism of acrylamide should be given in the introduction.

**R: We sincerely thank the Reviewer for this comment. We have now added this explanation in the Introduction section, as follows:**

***“Acrylamide is included by the International Agency for Research on Cancer into the group 2A, i.e., a probable human carcinogenic [6]. From a metabolic point of view, acrylamide can be metabolized using two major pathways. The first one is the pathway involving cytochrome P450 2E1 (CYP2E1)-mediated phase: through this way the oxidative metabolite glycidamide is consequently formed. Glycidamide can react with DNA to create DNA adducts. The second pathway of acrylamide metabolism is the direct conjugation with reduced glutathione (GSH). Acrylamide and glycidamide are also able to be associated with albumin or other plasma proteins. Both acrylamide and glycidamide can react with the N-terminal valine residues of Hemoglobin to form Hb adducts of acrylamide, that are widely used as biomarkers for acrylamide exposure [7].”***

There are typos in the paper. Some corrections are listed below.

ABSTRACT
Background:
• “Dietary acrylamide is present is several deep-frying, oven-baking, and roasting foods and associated with higher inflammatory and oxidative stress parameters.” Should be written as “Dietary acrylamide is found in certain foods, such as deep frying, baking and roasting, and is associated with higher inflammatory and oxidative stress parameters.”
Methods:
• “as increase in” should be written as “as an increase in”
• “using a linear regression” should be written as “using linear regression”
INTRODUCTION
• “Moreover, analogues” should be written as “Moreover, analogs”
• “glutathione (GSH) reduction.(6)” and “interleukin 6 (IL-6). (6)” reference (6) should be removed. In the continuation of these two sentences in the same paragraph “6th” reference has already been given.
METHODS
Exposure:
• “We divided the participants in gender-specific” should be written as “We divided the participants into gender-specific”
Outcomes:
• “20-meter at usual pace” should be written as “20-meter at a usual pace”
Covariates:
• “presence of knee OA” acronym should be written as full terminology in the first instance.
Statistical analysis:
• “we used a linear regression analysis.” Should be written as “we used linear regression analysis.”
RESULTS
Descriptive analyses:
• “by dietary acrylamide intake divided in”, “in” should be written as “into”
• “had a significant higher calorie intake”, “significant” should be written as “significantly”
• “but more physical active”, “physical” should be written as “physically”

Table 1:
“Charlson comoribidity index (points)”, “comoribidity” should be corrected as “comorbidity”

DISCUSSION
• “had a significant higher calorie intake”, “significant” should be written as “significantly”
• “indicating the need of future research”, “of” should be written as “for”

**R: Thank you so much for all these comments and for your careful reading. We have now corrected all these typos, as suggested.**

Reviewer #2: The link for the data is not working.

**R: Thank you for your careful reading. We have used a previous version of the website. In the Revised version, you can find the correct link.**

Only Dietary acrylamide intake was obtained through a food frequency questionnaire and correlated with the Physical performance. No other details regarding to the health parameters were shared or discussed. It would strengthen the results.

**R: We would like to sincerely thank the Reviewer. The results were already adjusted for the Charlson comorbidity index that included relevant health issues and medical conditions. However, we have reported the prevalence of some important medical conditions for better understanding the prevalence of some common diseases that could associated with dietary acrylamide and poor physical performance.**
